# Antibacterial activity of the novel oxazolidinone contezolid (MRX-I) against *Mycobacterium abscessus*

**DOI:** 10.3389/fcimb.2023.1225341

**Published:** 2023-08-15

**Authors:** Shan. Gao, Wenjuan. Nie, Lina. Liu, Lei. Su, Yingxia. You, Ruixue. Geng, Naihui Chu

**Affiliations:** ^1^ Tuberculosis Department, Beijing Tuberculosis and Thoracic Tumor Research Institute, Beijing, China; ^2^ Tuberculosis Department, Beijing Chest Hospital, Capital Medical University, Beijing, China; ^3^ Tuberculosis Department, Hengshui Third People’s Hospital, Hengshui, China; ^4^ Tuberculosis Department, Henan Province Anyang City Tuberculosis Prevention and Control Institute, Anyang, China; ^5^ Tuberculosis Department, Zhengzhou Sixth People’s Hospital, Zhengzhou, China; ^6^ Tuberculosis Department, Hohhot Second Hospital, Hohhot, China

**Keywords:** *Mycobacterium abscessus*, contezolid, zebrafish, activity, *in vivo*

## Abstract

**Objective:**

To evaluate contezolid (MRX-I) antibacterial activity against *Mycobacterium abscessus in vitro* and *in vivo* and to assess whether MRX-I treatment can prolong survival of infected zebrafish.

**Methods:**

MRX-I inhibitory activity against *M. abscessus in vitro* was assessed by injecting MRX-I into zebrafish infected with green fluorescent protein-labelled *M. abscessus*. Thereafter, infected zebrafish were treated with azithromycin (AZM), linezolid (LZD) or MRX-I then maximum tolerated concentrations (MTCs) of drugs were determined based on *M. abscessus* growth inhibition using one-way ANOVA. Linear trend analysis of CFU counts and fluorescence intensities (mean ± SE values) was performed to detect linear relationships between MRX-I, AZM and LZD concentrations and these parameters.

**Results:**

MRX-I anti-*M. abscessus* minimum inhibitory concentration (MIC) and MTC were 16 μg/mL and 15.6 μg/mL, respectively. MRX-I MTC-treated zebrafish fluorescence intensities were significantly lower than respective LZD group intensities (whole-body: 439040 ± 3647 vs. 509184 ± 23064, p < 0.01); head: 74147 ± 2175 vs. 95996 ± 8054, p < 0.05). As MRX-I concentration was increased from 0.488 μg/mL to 15.6 μg/mL, zebrafish whole-body, head and heart fluorescence intensities decreased. Statistically insignificant differences between the MRX-I MTC group survival rate (78.33%) vs. corresponding rates of the 62.5 μg/mL-treated AZM MTC group (88.33%, p > 0.05) and the 15.6 μg/mL-treated LZD MTC group (76.67%, p > 0.05) were observed.

**Conclusion:**

MRX-I effectively inhibited *M. abscessus* growth and prolonged zebrafish survival when administered to *M. abscessus*-infected zebrafish, thus demonstrating that MRX-I holds promise as a clinical treatment for human *M. abscessus* infections.

## 1 Introduction

Incidence and associated mortality rates of nontuberculous mycobacterial (NTM) lung disease are increasing worldwide (Cowman et al., 2019), thus highlighting the urgent need for improved NTM disease management. NTM is classified into fast-growing and slow-growing mycobacterial groups, with *Mycobacterium abscessus* the most commonly isolated fast-growing mycobacterial species associated with human NTM infections. According to 2020 NTM treatment guidelines prepared by the American Thoracic Society (ATS), the European Respiratory Society (ERS), the European Society of Clinical Microbiology and Infectious Diseases (ESCMID) and the Infectious Diseases Society of America (IDSA) (Daley et al., 2020). Moreover, the studies (Choi et al., 2012; Nie et al., 2014) have shown that azithromycin(AZM) has more antibacterial effect on Mycobacterium abscess both *in vivo* and *in vitro*, and it is not easy to develop resistance to macrolides. a multi-drug regimen containing macrolides is strongly recommended for the treatment of *M. abscessus* infection-associated lung disease. Nevertheless, the choice of effective therapeutic drugs is limited by the natural resistance of *M. abscessus* to commonly administered antibiotics (Nessar et al., 2012).

Linezolid (LZD), an oxazolidinone antibiotic that inhibits bacterial protein synthesis by binding to 30S and 50S ribosomal subunit rRNAs (Vinh and Rubinstein, 2009), was recommended as a treatment for *M. abscessus*-induced lung disease by the American Chest Association in 2007 and the British Chest Association in 2017 (Griffith et al., 2007; Haworth et al., 2017). In fact, LZD works well as a drug-resistant tuberculosis (TB) treatment (Conradie et al., 2020), as well as a treatment for improving rates of conversion of sputum *M. abscessus*-positive cultures to negative culture status for patients with pulmonary *M. abscessus* infection (Li et al., 2019). However, despite its efficacy, LZD use has been clinically limited, since the drug can trigger severe bone marrow suppression as a serious safety issue (Hashemian et al., 2018). Therefore, safer drugs are needed for TB and NTM disease treatment. Contezolid (MRX-I) is a novel oxazolidinone antibiotic with the chemical name (S)-5-[(isoxazole-3-arylamino)methyl]-3-[2,3,5-trifluoro-4-[4-oxo-3,4-dihydropyridine-1(2H)-yl]phenyl]oxazolidine-2-one (Gordeev and Yuan, 2014) that provides the same anti-*M. tuberculosis* effect as LZD (Shoen et al., 2018). Nonetheless, despite the fact that MRX-I treatment is associated with markedly reduced rates of bone marrow suppression and monoamine oxidase inhibition (MAOI) rates than those reported for LZD (Cowman et al., 2016), few studies have investigated *M. abscessus* susceptibility and resistance to MRX-I, prompting this study. Here, antibacterial effects of MRX-I treatment were assessed both *in vitro* and *in vivo* using the *M. abscessus* standard strain. Furthermore, the efficacy of the drug for prolonging survival was also assessed using *M. abscessus*-infected zebrafish as an *in vivo* bacterial infection model.

## 2 Materials and methods

### 2.1 Minimum inhibitory concentration determinations

AZM and LZD are both purchased by Beijing Solarbio Science & Technology Co., Ltd.(Beijing, China), and MRX-I is provided by Shanghai Micurx Pharmaceutical Co., Ltd. (Shanghai, China). Both drugs were dissolved in dimethyl sulfoxide (DMSO), and the drug solution was prepared according to the suggestions provided by the Institute of Clinical and Laboratory Standards (CLSI) (Woods et al., 2011). The standard strain of M. abscessus ATCC 19977 was cultured on solid Lowenstein-Jensen medium at 37°C for 4-6 days. The MICs of AZM, LZD and MRX-I were determined by adding drugs to M. abscessus cultured in 96-well plates according to the recommended CLSI broth microdilution method. The broth was diluted twice, and both the concentrations of AZM, LZD and MRX-I ranged from 0.5-256μg/mL. A bacterial inoculum with turbidity equivalent to 0.5 McFarland standard dilution of 1∶200 was prepared for each strain. The MIC of *M. abscessus* was determined after 3 days of culture at 37°C together with antibiotics. Thereafter, 70 μl of Alamar Blue solution (Sirotec, 20μL Alamar Blue+50μL 5% Tween 80) was added to each well, and then the plates were incubated for another 24 hours. The color change from blue to pink indicates bacterial growth (Cowman et al., 2016). MIC is defined as the lowest drug concentration that no color change, that is, the lowest concentration that can inhibit the visible growth of the test isolate. Explain the results of drug sensitivity test (DST) according to the breakpoint recommended by CLSI.

### 2.2 Microinjection of *M. abscessus* into zebrafish as an *in vivo* bacterial infection model

This study was approved by the Ethics Committee of Beijing Chest Hospital, a hospital affiliated with Capital Medical University (2021-020). The wild-type zebrafish AB strain was maintained through natural paired mating to generate zebrafish progeny that were raised in water at 28 °C. Meanwhile, the smooth (S) morphology *M. abscessus* standard strain (ATCC19977) was incubated for 5 to 7 days at 30 °C in Middlebrook 7H9 broth (Becton Dickinson) containing 10% OADC (Becton Dickinson) and 0.05% Tween 80 (Sigma-Aldrich). Mid-log-phase *M. abscessus* cultures were centrifuged then the pellets were washed and cells were resuspended in phosphate buffered saline (PBS) containing 0.05% Tween 80. Next, the bacterial suspension was homogenised and sonicated then the tube containing dispersed cells was left upright for 5 to 10 min while bacterial cells settled to the bottom of the tube. The bacteria were then collected and resuspended in a smaller volume of PBS and labelled with the green fluorescent dye DIO (Thermo Fisher Scientific, USA or Hill Technology Co., Ltd., China). Labelled bacteria were next introduced into wild-type zebrafish at 2 days post-fertilisation (dpf) by micro-intravenous injection of about 3.6 × 10^3^ colony-forming units (CFUs) into the tail of each zebrafish in order to establish a zebrafish *M. abscessus* infection model. Zebrafish were anesthetized using 3-aminobenzoic acid ethyl ester methanesulfonate (C_9_H_11_NO_2_·CH_4_O_3_S, MESAB). The MESAB was prepared by mixing MESAB and Na_2_HPO_4_·12H_2_O in a total mass ratio of 1:5 to make a 4 mg/mL solution, which was stored at 4°C. For use, it was diluted with standard dilution water, with a final anesthetic concentration of 0.16 mg/mL. Then the zebrafish were injected bacteria by intravenous microinjection with each fish receiving approximately 3.6×10³colony forming units (CFUs)of the transplant to establish the zebrafish model of M. abscessus infection (Guo et al., 2020; Nie et al., 2020).

### 2.3 Maximum tolerated concentrations of AZM, LZD and MRX-I in zebrafish

Zebrafish collected at 3 dpf under the microscope were randomly allocated to wells of 6-well plates (30 zebrafish per well). Meanwhile, azithromycin (AZM), LZD and MRX-I were prepared in dimethylsulfoxide (DMSO, Shanghai Aladdin Biochemical Technology Co., Ltd.) as initial 20.0 mg/mL stock solutions that were stored at -20 °C. For MTC determinations, drugs were diluted in water then were added to wells containing zebrafish to generate dilutions in wells of 6-well plates at final concentrations for AZM (62.5 μg/mL, 125 μg/mL, 250 μg/mL, 500 μg/mL, 1000 μg/mL), for LZD (15.6 μg/mL, 31.2 μg/mL, 62.5 μg/mL, 125 μg/mL, 250 μg/mL) and for MRX-I (15.6 μg/mL, 31.2 μg/mL, 62.5 μg/mL, 125 μg/mL, 250 μg/mL). The blank control group (zebrafish without injected *M. abscessus* and without drug dosing) and the model group (the negative control group of zebrafish injected with *M. abscessus* without drug dosing) were prepared at the same time using the same methods used to prepare the abovementioned drug-treated samples. After plates were incubated for 48 h at 35 °C, MTCs were determined as based on the highest concentration of each drug that did not cause zebrafish death.

### 2.4 Evaluation of *in vivo* MRX-I efficacy against *M. abscessus* using the *M. abscessus*-infected zebrafish model

According to previously reported experimental procedures, 3 dpf zebrafish were selected under the microscope then were randomly assigned to 6-well plates (30 zebrafish/well). Initial AZM, LZD and MRX-I stock solutions were diluted in water to generate working solutions of AZM (62.5 μg/mL), of LZD (15.6 μg/mL) and of MRX-I (0.488 μg/mL, 0.977 μg/mL, 1.95 μg/mL, 3.91 μg/mL, 7.81 μg/mL, 15.6 μg/mL). The control group and the model group were concurrently set up. All control and experimental samples were prepared in the same final volume of 3 mL/well. After treatment of zebrafish at 35 °C for 48 h, 10 zebrafish were randomly selected from each experimental group to photograph. The zebrafish were anesthetized and transferred onto methyl cellulose using a Pasteur pipette. An electrically controlled, continuously variable magnification fluorescence microscope (AZ100, Nikon, Japan) equipped with a green fluorescence channel was used at a magnification of 30x. Image processing software (NIS-Elements D 3.20) was used to analyse and collect fluorescence-based data. The efficacy of each drug, as based on inhibition of *M. abscessus* growth in zebrafish, was evaluated as based on fluorescence intensity of zebrafish whole-body, head and heart tissues.

### 2.5 Evaluation of MRX-I efficacy for prolonging survival of zebrafish infected with *M. abscessus*

3 dpf zebrafish were selected under the microscope and randomly assigned to 50-mL beakers (cups) with 60 zebrafish (experimental group) in a 20-mL volume per cup. Initial AZM, LZD and MRX-I stock solutions were diluted to generate a 62.5-μg/mL AZM MTC solution, a 15.6-μg/mL LZD MTC solution and MRX-I solutions at concentrations of 0.488 μg/mL, 0.977 μg/mL, 1.95 μg/mL, 3.91 μg/mL, 7.81 μg/mL and 15.6 μg/mL (MTC). Concurrently, control group and model group samples were set up in 20-mL volumes in cups then all control and experimental group cups were incubated at 35 °C. Numbers of zebrafish deaths for all groups were recorded every day and dead zebrafish were removed daily. At experiment completion, data were statistically analysed to calculate survival rates of zebrafish in each experimental and control group.

### 2.6 Data analysis

SPSS 26.0 was used to statistically analyse all data obtained in this study. One-way ANOVA was used for intergroup comparisons of CFU counts and fluorescence intensity expressed as mean ± SE values. For comparisons between two groups, t or t’ tests were used to compare normally distributed data from independent samples and non-parametric tests were used to compare data with non-normal distributions. One-way ANOVA analysis was chosen to compare normally distributed data with equal variance among multiple groups; otherwise non-parametric tests were used. Linearity between MRX-I concentration and fluorescence intensity was assessed using a linear trend analysis method. Kaplan-Meier survival analysis was performed using the log-rank test to visualise survival rates of zebrafish treated with different MRX-I concentrations. Intergroup differences with p values of <0.05 were considered statistically significant.

## 3 Results

### 3.1 Effects of AZM, LZD and different MRX-I concentrations on *M. abscessus* growth in zebrafish

MICs of AZM, LZD and MRX-I were determined according to their effects on growth of the *M. abscessus* standard strain in zebrafish. The MIC of MRX-I for inhibition of growth of the *M. abscessus* standard strain was 16 μg/mL, and the MICs of AZM and LZD were 0.5µg/mL and 8μg/mL, respectively.

**Table 1** presents results related to efficacies of AZM, LZD and MRX-I against *M. abscessus* infection. The AZM MTC was 62.5 µg/mL and MTCs of LZD and MRX-I were both 15.6 µg/mL. Treatment of zebrafish with AZM at 2 times the MTC led to deterioration of zebrafish health; treatment with 8 times the MTC led to signs of cardiac congestion accompanied by body bending; treatment with 16 times the MTC led to zebrafish mortality approaching the rate of 33.33%. At the LZD MTC of 15.6 μg/mL, no notable zebrafish health differences were observed as compared to that of the model group, while LZD treatment of zebrafish at 2 times the MTC resulted in a dramatic increase in mortality rate to 23.33%. Similarly, treatment with MRX-I led to similar trends as those observed for LZD; at the MRX-I MTC of 15.6 μg/mL, no significant changes were observed in zebrafish phenotype as compared with that of the model group, while treatment with 2 times the MRX-I MTC led to a marked increase in mortality rate.

**Table 1:** The MTC of AZM, LZD and MRX-I in zebrafish in vivo.

Groups	concentration (μg/mL)	number of deaths (n)	mortality rate (%)	phenotype
Control(n = 30)	–	0	0	normal
Model(n = 30)	–	0	0	normal
AZM(n = 30)	62.5	0	0	similar to model group
	125	0	0	5 body bent
	250	1	3.33	3 body bent
	500	1	3.33	1 heart congestion,5 body bent
	1000	10	33.33	–
LZD(n = 30)	15.6	0	0	similar to model group
	31.2	7	23.33	–
	62.5	8	26.67	–
	125	9	30.00	–
	250	9	30.00	–
MRX-I(n = 30)	3.9	0	0	similar to model group
	7.8	0	0	similar to model group
	15.6	0	0	similar to model group
	31.2	5	16.67	–
	62.5	5	16.67	–
	125	15	50.00	–
	250	20	66.67	–

### 3.2 Evaluation of efficacies of AZM, LZD and MRX-I against *M. abscessus* in the zebrafish bacterial infection model

**Figure 1:**
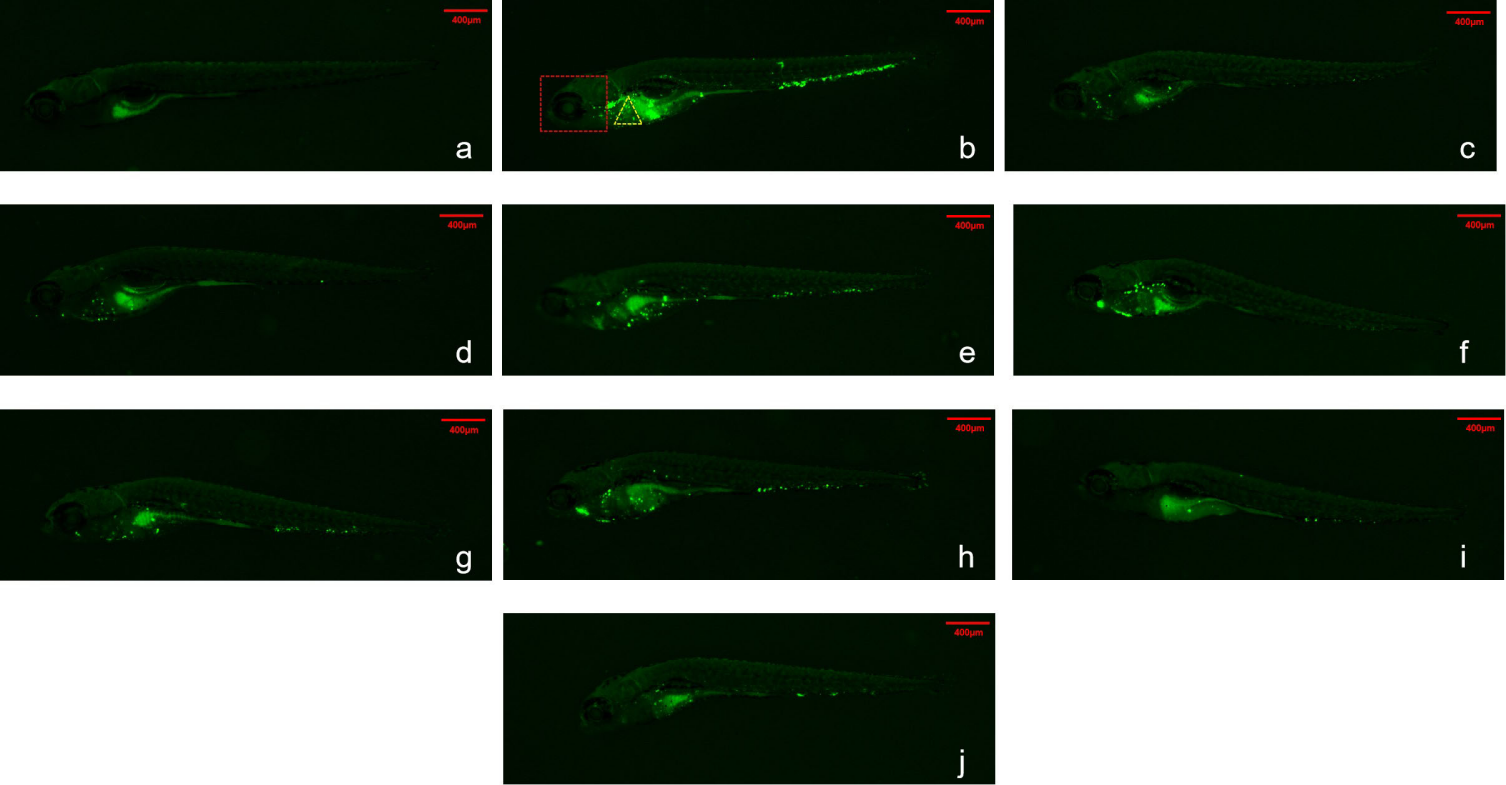
Fluorescence intensity distribution of different drugs in zebrafish. Green spots are Mycobacterium abscessus. The red dotted box represents the head analysis area. The yellow dotted box represents the heart analysis area. **(A)** Control; **(B)** Model; **(C)** 62.5μg/mL AZM; **(D)** 15.6μg/mL LZD; **(E)** 0.488μg/mL- MRX-I; **(F)** 0.977μg/mL-MRX-I; **(G)** 1.95μg/mL-MRX-I; **(H)** 3.91 μg/mL- MRX-I; **(I)** 7.81μg/mL- MRX-I; **(J)** 15.6 μg/mL-MRX-I.

**Figure 2:**
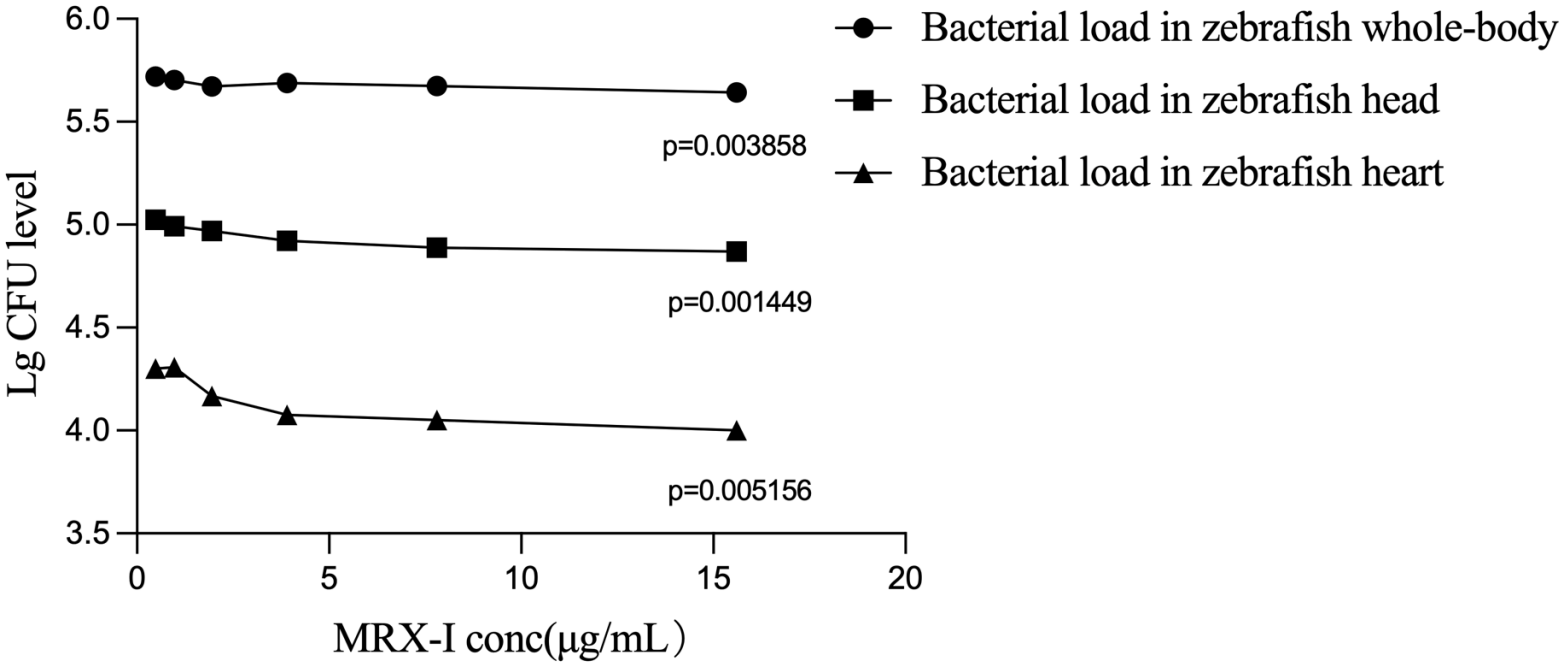
Therapeutic effect of different concentrations of WX-081 on M. abscessus infection in zebrafish whole-body, head and heart. With the increase of MRX-I concentration, the level of CFU in zebrafish gradually decreased, and the linear trend of each group was P < 0.05.

Fluorescence intensities of zebrafish whole-body, head and heart tissues of AZM MTC, LZD MTC and MRX-I MTC groups were lower than that of the model group (**Table 2**). The LZD MTC group whole-body fluorescence intensity exceeded that of the MRX-I MTC group (509184 ± 23064 vs. 439040 ± 3647, p < 0.01). Comparisons of effects of different MRX-I concentrations revealed that the whole-body fluorescence intensity of the MRX-I MTC group was only lower than fluorescence intensities of the 1/32, 1/16 and 1/4 MTC MRX-I groups (439040 ± 3647 vs. 524203 ± 31487, P < 0.01; 439040 ± 3647 vs. 505230 ± 16923, p < 0.01; 439040 ± 3647 vs. 487036 ± 11374, P < 0.01, respectively), with zebrafish whole-body fluorescence intensity decreasing with increasing MRX-I concentration (p = 0.004). Zebrafish head fluorescence intensities were lower in the MRX-I MTC group than in the MRX-I 1/32 MTC group (74147 ± 2175 vs. 105710 ± 10573, P < 0.05), the MRX-I 1/8 MTC group (74147 ± 2175 vs. 93451 ± 5928, P < 0.01) and the MRX-I 1/4 MTC group (74147 ± 2175 vs. 83397 ± 3571, P < 0.05), while the head fluorescence intensity of the LZD MTC group was higher than that of the MRX-I MTC group (95996 ± 8054 vs. 74147 ± 2175, P < 0.05). As for whole-body fluorescence intensity, head fluorescence intensity decreased with increasing MRX-I concentration (p = 0.001). By contrast, the MRX-I MTC group zebrafish heart fluorescence intensity was lower than corresponding intensities of the MRX-I 1/16 MTC and 1/8 MTC groups (10011 ± 852 vs. 20258 ± 4171, P < 0.05; 10011 ± 852 vs. 14733 ± 1663, P < 0.05, respectively), with zebrafish cardiac fluorescence intensity increasing linearly with increasing MRX-I concentration (p = 0.005). **Figure 1** shows zebrafish fluorescence intensity distributions for different tissues of zebrafish treated with different concentrations of the three drugs.Increased WX-081 concentration was associated with lower bacterial burdens in both whole-body, head and heart of zebrafish as determined by bacterial CFU (**Figure 2**).

**Table 2:** The efficacy of AZM, LZD, and MRX-I against Mycobacterium Abscess infection in zebrafish in vivo.

Groups	concentration (μg/mL)	Whole-body fluorescence intensity (pixel, mean ± SE)	Head fluorescence intensity (pixel, mean ± SE)	Heart fluorescence intensity (pixel, mean ± SE)
Control (n = 10)	–	361707±7139***	55473±2670***	3854±158***
Model (n=10)	–	671089±22305	132412±9989	22310±1729
AZM (n = 10)	62.5	433684±11910***	78397±4815***	11847±1431***
LZD (n = 10)	15.6	509184±23064***^a2^	95996±8054*^a1^	11034±2054**
MRX-I (n=10)	0.488	524203±31487^a2^	105710±10573^a1^	19992±4702
	0.977	505230±16923^a2^	98355±10939	20258±4171^a1^
	1.95	470785±14787**	93451±5928^a2^	14733±1663^a1^
	3.91	487036±11374*^a2^	83397±3571*^a1^	11890±854*
	7.81	472309±22530***	77426±4692***	11259±811*
	15.6	439040 ± 3647***	74147±2175***	10011±852***

Compared with the model group, *p < 0.05, **p < 0.01, ***p < 0.001.

Compared with 15.6 μg/mL MRX-I, ^a1^p < 0.05, ^a2^p < 0.01.

### 3.3 Evaluation of MRX-I effect on survival time of model zebrafish infected with *M. abscessus*

**Figure 3:**
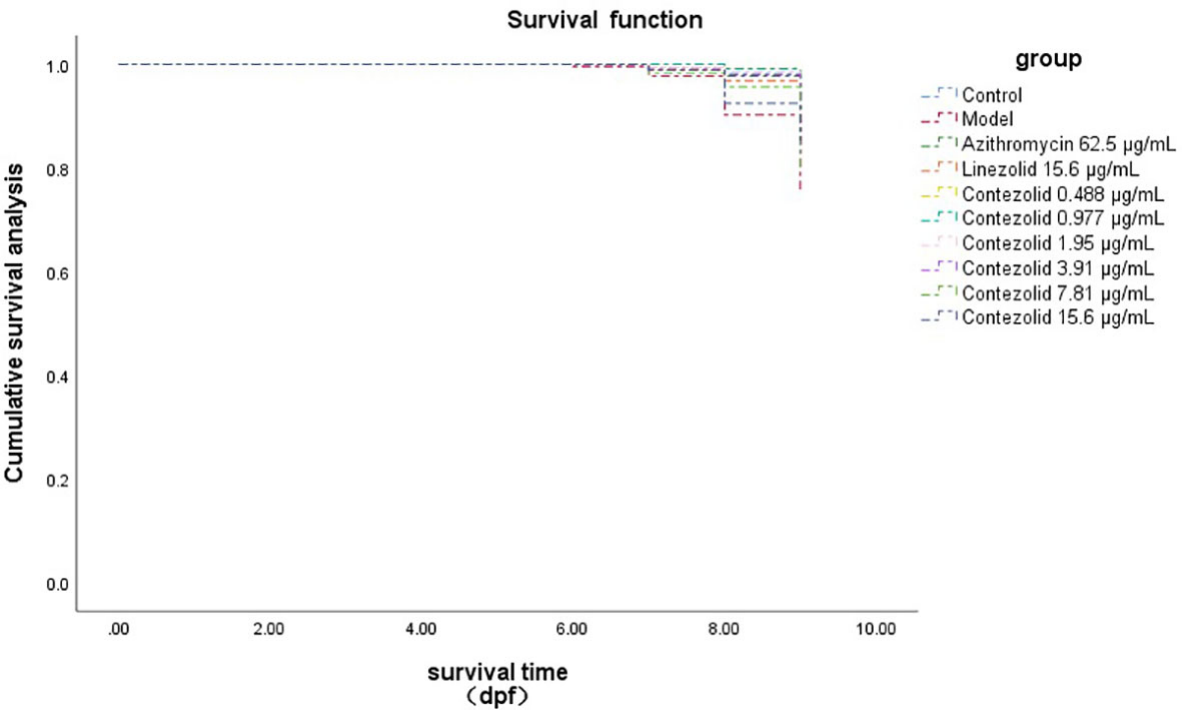
Kaplan-Meier survival curves for zebrafish with different drugs.

The survival rate of zebrafish in the MRX-I MTC group was 78.33%, a rate that was not significantly different from survival rates of MRX-I 1/16, 1/8, 1/4 and 1/2 MTC groups (**Table 3**). In addition, the survival rate of the MRX-I MTC group was not significantly different from that of the AZM MTC group that was treated with 62.5 μg/mL (78.33% vs. 88.33%, p > 0.05) and that of the LZD MTC group treated with 15.6 μg/mL (78.88% vs. 76.67%, p > 0.05). As compared with the model group survival rate, no significant difference in LZD and MRX-I MTC (15.6 μg/mL) group survival rates were observed (66.67% vs. 76.67%, p > 0.05; 66.67% vs. 78.33%, p > 0.05, respectively), although MRX-I 1/32, 1/16 and 1/8 MTC group survival rates exceeded that of the model group (95.00% vs. 66.67%, p < 0.001; 90.00% vs. 66.67%, p < 0.01; 85.00% vs. 66.67%, p < 0.05, respectively). **Figure 3** shows zebrafish survival curves for different concentrations of AZM, LZD and MRX-I.

**Table 3:** Effect of AZM, LZD and different concentrations of MRX-I of survival in zebrafish.

Groups	concentration(μg/mL)(μg/mL)	4-9 dpf survival rate(%)
Control(n = 60)	–	91.67^b3c1^
Model(n = 60)	–	66.67
AZM(n = 60)	62.5	88.33^b2^
LZD(n = 60)	15.6	76.67
MRX-I(n = 60)	0.488	95.00^b3c2^
	0.977	90.00^b2^
	1.95	85.00^b1^
	3.91	80.00
	7.81	75.00
	15.6	78.33

Compared with the model group, ^b1^p < 0.05, ^b2^p < 0.01, ^b3^p < 0.001.

Compared with 15.6 μg/mL MRX-I, ^c1^p < 0.05, ^c2^p < 0.01.

## 4 Discussion

Zebrafish, a small (<4-cm-long) freshwater fish, is used as an animal model, due to similarities of its innate and acquired immune systems with those of mammals that render it more advantageous for researching human diseases than invertebrate models (e.g., *Drosophila melanogaster*, nematodes (van der Sar et al., 2004)). In recent years, an increasing number of studies (Bernut et al., 2014; Dupont et al., 2017; Lefebvre et al., 2017; Winters et al., 2022) have employed the *M. abscessus*-infected zebrafish model to assess antibacterial activities of drugs *in vivo*.

Macrolide drugs are one of the reliable drugs to treat *M. abscessus* infection, but it is also easy for *M. abscessu* to produce macrolide drug-acquired mutations (Richard et al., 2020; Griffith and Daley, 2022). LZD is the first oxazolidinone used in humans, and it has anti-mycobacterium tuberculosis activity *in vitro* and *in vivo (* Shoen et al., 2018), meanwhile,it is also an effective drug for treating *M. abscessus* infection (Zhang et al., 2018), however, due to drug adverse reactions, its clinical practice is limited (Shoen et al., 2018). Here we explored the antibacterial activity of MRX-I, a new oxazolidinone antibiotic, against *M. abscessus in vitro* and *in vivo*. Previously, the *in vitro* activity of MRX-I against *M. abscessus* was studied by Guo et al., who demonstrated that *in vitro* MRX-I MICs within the range of 0.25 to 64 mg/L could serve as indicators of antibacterial activity against *M. abscessus*. However, no *in vivo* studies conducted using animal models infected with *M. abscessus* have been reported, prompting this study. Here we report the first *in vivo* investigation of the antibacterial activity of MRX-I, as assessed based on the drug’s effect in prolonging survival of *M. abscessus*-infected zebrafish. Our results revealed that the MRX-I MIC based on inhibition of *M. abscessus* growth in zebrafish was 16 μg/mL and the MRX-I MTC was 15.6 μg/mL. In addition, based on the fact that the main pathogenic subspecies isolated from human pathogenic *M. abscessus* strains are S-type/Smooth-type (Nie et al., 2014), as the concentration of MTX concentration was increased from 0.488 μg/mL to the MTC, whole-body, head and heart S-type *M. abscessus* fluorescence intensities gradually decreased. MRX-I and LZD showed similar anti-mycobacterium tuberculosis ability in mouse model (Shoen et al., 2018). However, in zebrafish model, when both MRX-I and LZD were in MTC concentration, the whole body fluorescence intensity of MRX-I group was lower than that of LZD group (439040 ± 3647 vs. 509184 ± 23064, p < 0.01), indicating that the anti-mycobacterium abscess activity of MRX-I might be better than LZD.

Notably, the survival rate of 4-9 dpf zebrafish treated with the MRX-I MTC was 78%, a rate that was lower than survival rates of the 1/32 MTC group and not statistically significantly different from survival rates of groups treated with other MRX-I concentrations. Moreover, fluorescence intensities of zebrafish whole-body, head and heart tissues were found to increase in a linear manner with increasing MRX-I concentration within the range of 0.488 μg/mL to 15.6 μg/mL. Therefore, a lower zebrafish fluorescence intensity reflected a stronger inhibitory effect on *M. abscessus* growth. Furthermore, the survival of 4-9 dpf zebrafish decreased from 95.00% to 78.33% as the concentration of MRX-I was increased from 0.488 μg/mL to the MTC. Taken together, the abovementioned results obtained using the *M. abscessus*-infected zebrafish model demonstrated that MRX-I may inhibite *in vivo* growth of *M. abscessus*.

Importantly, LZD can penetrate the blood-brain barrier and thus has good activity against intracranial infections. In order to verify whether MRX-I also has good intracranial antibacterial activity, head bacterial counts were specifically studied here. Our results revealed that *in vivo* bacterial counts in zebrafish heads decreased as MRX concentration was increased. Moreover, we investigated MRX-I effects on head fluorescence intensity of *M. abscessus* within zebrafish heads, whereby analysis of head fluorescence results revealed a lower head fluorescence intensity of the MRX-I MTC group as compared to those of the model group (p < 0.001) and groups treated with MRX-I at 1/8 (p < 0.01) and 1/4 (p < 0.05) of the MTC. Furthermore, as the drug concentration was gradually increased, head fluorescence intensity gradually decreased and the head bacterial count decreased linearly with increasing drug concentration, thus suggesting that MRX-I may reduce the bacterial load in zebrafish. Similarly, results of a previous study had suggested that LZD freely entered the cerebrospinal fluid of pulmonary TB patients with intact blood-brain barrier function (Nau et al., 2010). A case report of MRX-I in the treatment of tuberculous meningitis (Guo et al., 2023) shows that MRX-I can penetrate into cerebrospinal fluid well, as similar results of the current study indicating that MRX-I can act on M. abscessus in zebrafish head tissues. Nonetheless, clinical studies are needed to determine whether MRX-I can serve as an effective treatment for central nervous system TB cases.

This study had several limitations. First, here we used a zebrafish model of bacterial infection to assess MRX-I bacteriostasis against a single standard *M. abscessus* strain without studying the drug’s effects on other *M. abscessus* complex strains, including *M. abscessus* subspecies *massiliense* and *bolletii*. Second, the number of clinical isolates of *M. abscessus* analysed in this study was limited and thus should expanded in future studies to provide more reliable real-world data to help clinicians choose the most effective and safe drug regimens for patient treatment.

In conclusion, MRX-I (contelozid) exerts an inhibitory effect on *M. abscessus* growth and tends to prolong survival time of *M. abscessus*-infected zebrafish. Thus, results presented here should provide significantly relevant information to guide the clinical application of contezolid as a treatment for *M. abscessus* infection.

## Data availability statement

The original contributions presented in the study are included in the article/supplementary material. Further inquiries can be directed to the corresponding author.

## Ethics statement

The animal study was reviewed and approved by the Ethics Committee of Beijing Chest Hospital, Capital Medical University.

## Author contributions

SG and WN contributed to the experimental operation and article writing. LL, LS, YY, RG contributed to the experimental operation. NC contributed to the revision of the article. All authors contributed to the article and approved the submitted version.
